# Diffusion and Chemical Degradation of Vitamin B6 in Chickpeas (*Cicer arietinum* L.) during Hydrothermal Treatments: A Kinetic Approach

**DOI:** 10.3390/foods13121847

**Published:** 2024-06-12

**Authors:** Heba Shaban, Claus Kadelka, Stephanie Clark, Nicolas Delchier

**Affiliations:** 1 Department of Food Science and Human Nutrition, Iowa State University, 536 Farm House Ln, Ames, IA 50011, USA; hmshaban@iastate.edu (H.S.); milkmade@iastate.edu (S.C.); 2 Department of Mathematics, Iowa State University, 411 Morrill Rd., Ames, IA 50011, USA; ckadelka@iastate.edu

**Keywords:** pyridoxal, Fick’s law, leaching, thermal degradation, legumes, processing

## Abstract

Chickpeas are more sustainable than other food systems and have high a nutritional value, especially regarding their vitamin composition. One of the main vitamins in chickpeas is vitamin B6, which is very important for several human metabolic functions. Since chickpeas are consumed after cooking, our goal was to better understand the role of leaching (diffusion) and thermal degradation of vitamin B6 in chickpeas during hydrothermal processing. Kinetics were conducted at four temperatures, ranging from 25 to 85 °C, carried out for 4 h in an excess of water for the diffusion kinetics, or in hermetic bags for the thermal degradation kinetics. Thermal degradation was modeled according to a first-order reaction, and diffusion was modeled according to a modified version of Fick’s second law. Diffusivity constants varied from 4.76 × 10^−14^ m^2^/s at 25 °C to 2.07 × 10^−10^ m^2^/s at 85 °C; the temperature had an impact on both the diffusivity constant and the residual vitamin B6. The kinetic constant ranged from 9.35 × 10^−6^ at 25 °C to 54.9 × 10^−6^ s^−1^ at 85 °C, with a lower impact of the temperature. In conclusion, vitamin B6 is relatively stable to heat degradation; loss is mainly due to diffusion, especially during shorter treatment times.

## 1. Introduction

Legumes are a type of plant belonging to the *Fabaceae* family that includes a large variety of peas, beans, lentils, and peanuts. All legumes are annual crops of varying sizes, shapes, and colors, and are commonly used for food and feed [1]. Pulses are a specific category of legumes that refers to their dried seeds. Common pulses include lentils, dry peas, various types of beans (such as black, kidney, and navy beans), and chickpeas. Legumes appear to be more efficient in terms of sustainability than other food systems, such as meat, due to their lower water requirement and ability to fix nitrogen in soil. At the same time, legumes have high nutritional value based on their protein content, as well as essential amino acids, starch, and minerals.

Chickpeas (*Cicer arietinum* L.) are widely consumed worldwide, contributing a good source of macro and micronutrients. Dry chickpeas contain, on average, 20.5 g/100 g of protein, 12.2 g/100 g of fiber (8.9 g/100 g and 7.6 g/100 g in cooked chickpeas, respectively), and several minerals (i.e., 4.3 mg/100 g and 2.9 mg/100 g of iron in dry and cooked, respectively) [2]. Dry chickpeas are also rich in vitamins, with an average content of 4.0 mg/100 g of vitamin C (1.3 mg/100 g in cooked), 1.55 mg/100 g of niacin (0.53 mg/100 g in cooked), and 0.56 mg/100 g of folates (0.17 mg/100 g in cooked). Chickpeas are also a good source of vitamin B6, with its concentration in raw material ranging from 0.39 to 0.79 mg/100 g (dry weight—dw) 0.53 mg/100 g average in raw and 0.14 mg/100 g in cooked chickpeas [2,3,4,5,6,7,8].

Vitamin B6 is important for health because it is required for several metabolic reactions to synthesize carbohydrates, lipids, amino acids, and nucleic acids synthesis. Deficiencies in this vitamin have been associated with several physiological disorders and diseases, such as inflammation, cancers, cardiovascular diseases, atherosclerosis, and neurological disorders. The National Institutes of Health in the USA (https://ods.od.nih.gov/factsheets/VitaminB6-Consumer/, accessed on 1 September 2023) suggests a Recommended Dietary Allowance (RDA) of 1.3 mg per day for men and women (from 19 to 50 years); this increases to 1.7 mg per day for men and 1.5 mg per day for women over 50. The RDA increases to 1.9–2.0 mg during pregnancy and lactation. In the UK, the National Health Service (https://www.nhs.uk/conditions/vitamins-and-minerals/vitamin-b/, accessed on 1 September 2023) recommends a daily intake of 1.4 mg for men and 1.2 mg for women. In Europe, the EFSA (https://www.efsa.europa.eu/fr/efsajournal/pub/4485, accessed on 1 September 2023) set up Population Reference Intake as 1.7 mg per day for men and 1.6 mg per day for women, increasing to 1.8 mg per day during pregnancy. The World Health Organization/Food and Agriculture Organization set a Recommended Nutrient Intake (RNI) of 1.3 mg daily for adults aged 19–50 [9]. Around 23% of the elderly population in Europe, 11 to 65% in the UK, and 14 to 49% in the USA (15 to 23% for men and 14 to 49% for women) have a vitamin B6 deficiency [10,11,12,13].

Vitamin B6 is represented by different vitamers, including pyridoxal, pyridoxamine, pyridoxine, their phosphorylated forms, and a glycosylated form of pyridoxine. The three forms of the vitamin are present in chickpeas with a high proportion of pyridoxine [14]. The glycosylated form of vitamin B6 is about 0.37 mg/100 g [7]. Although chickpeas are a good source of vitamin B6, they are not consumed raw, but mainly after soaking and/or cooking in water (i.e., boiled or pressure-cooked). Hydrothermal treatments cause physicochemical changes that affect the stability of the vitamin [15]. The concentration of vitamin B6 has been reported to range from 0.047 mg/100 g after canning (circa 87% loss) to 0.267 mg/100 g after boiling (about 30% loss) [3,16,17,18]. Similarly losses of vitamin B6 due to cooking beef vary by cooking method, with the greatest losses observed when beef is braised (45 to 70%) or stewed (50 to 75%), rather than grilled (5 to 30% loss) [19].

The mechanisms underlying the losses of water-soluble vitamins in fruits and vegetables have predominantly focused on vitamin C and folates, leading to a better understanding of the relative contribution of diffusion and thermal degradation on the total loss [20,21]. Few studies have been carried out for vitamin B6. Thermal degradation was studied in model systems or food systems (broccoli or bread), with reactions following a first-order or pseudo-first-order kinetics, with activation energies (Ea) varying from 87 × 10^3^ J/mol to 115 × 10^3^ J/mol, and rate constants (k) ranging from 6 × 10^−4^ min^−1^ to 324 × 10^−3^ min^−1^ depending on the studied matrix [22,23,24,25]. Diffusion of vitamin B6 was only studied using a gelatine agarose gel system at 23 °C, yielding diffusivity constants of 8.9 × 10^−14^ m^2^/s and 4.4 × 10^−13^ m^2^/s, depending on the gelatine agarose ratio [26].

When cooking vegetables in water, two loss mechanisms co-occur for water-soluble vitamins: thermal degradation and diffusion (leaching) into the surrounding cooking water [20]. This work aims to assess and integrate the relative importance of these two physicochemical mechanisms for vitamin B6 in chickpeas. It was hypothesized that diffusion predominantly governs the initial phase of vitamin loss, as vitamins migrate from the chickpeas’ matrix into the surrounding medium. As the treatment progresses and temperatures rise, thermal degradation becomes the primary mechanism contributing to further vitamin loss. The relative contributions of these processes vary depending on the treatment temperature and duration, with higher temperatures accelerating thermal degradation. For this reason, vitamin B6 content during hydrothermal treatment was assessed in two experiments: one that only allowed degradation (heating in hermetically closed bags), and another that allowed degradation and leaching (diffusion in a large excess of water). Both experiments were performed at the same temperatures to determine the net contribution of the diffusion to overall vitamin B6 loss (diffusion and degradation). Chickpeas were chosen due to their relatively high vitamin B6 content, broad consumption and sustainability characteristics, and spherical shapes, enabling relatively simple diffusion modeling using Fick’s second law.

## 2. Materials and Methods

### 2.1. Chemicals

Methanol HPLC grade and phosphoric acid were purchased from Fisher Scientific (Fair Lawn, NJ, USA). Pyridoxal hydrochloride, pyridoxine hydrochloride with purity > 98%, sodium borohydride, acetic acid, and β-glucosidase (3 U/mg) were purchased from Sigma Aldrich (St Louis, MO, USA). Glyoxylic acid monohydrate and sodium 1-heptanesulfonic acid were purchased from TCI (Portland, OR, USA). Acid phosphatase extracted from potato (Grade II, 2 U/mg) was purchased from Roche Molecular Biochemicals (Indianapolis, IN, USA).

### 2.2. Plant Material and Samples Preparation

Dried bulk chickpeas (*Cicer arietinum* L.) were purchased (on batch for each set of experiments) from a local supermarket (Wheatsfield, Ames, IA, USA) and stored in a dark, dry environment until kinetics experiments were carried out (no longer than three months).

Thermal kinetics and diffusion kinetics were performed as described by Renard et al. [21]. Chickpeas were boiled in a stockpot (lid on), with a solid-to-liquid ratio of 335 g to 1 L, for 30 min. They were directly drained and placed into the shaking water bath (Grant LSB Aqua pro, VWR) to start the kinetics experiment. Diffusion kinetics were carried out for 4 h, with a solid-to-liquid ratio of 50 g to 1 L, under aerobic conditions, and temperature-controlled at 25, 45, 65, and 85 ± 0.1 °C. The lid of the water bath was covered with aluminum foil, and the shaking speed was set to 150 rpm. For the thermal degradation experiments, chickpeas were firstly boiled in the same conditions as the diffusion kinetics and drained; then, 50 g of material was quickly placed into single hermetic plastic bags (resalable plastic bags with zipper—15 × 23 cm, Amazon.com, Inc., Seattle, WA, USA) to avoid leaching of the vitamins. Each bag, corresponding to an individual sampling point, was placed into the shaking water bath to start the kinetics. Kinetics were performed in the same conditions as the diffusion ones (shaking, temperature, aerobic conditions). Chickpeas were collected, drained (for diffusion kinetics) at each sampling time, and then directly stabilized by freezing in liquid nitrogen. Samples were ground in liquid nitrogen and stored at −20 °C, until analysis. Three full replicates (different batches on different days) were carried out for each condition (diffusion and thermal degradation).

### 2.3. Total Vitamin B6 Quantification

Total vitamin B6 content (expressed as pyridoxine) was determined according to the AOAC method with few modifications [27]. Briefly, extraction was carried out with 3.0 g of chickpea powder, homogenized with a mixture composed of 2 mL of sodium acetate solution (0.625 mol/L), 2.5 mL of glyoxylic acid solution (1 mol/L), 0.8 mL of ferrous sulfate solution (10 g/L), 1 mL of acid phosphatase solution (20 mg/mL), and 1.0 mL of 15 mg/mL β-glucosidase. The mixture was placed in a shaking incubator (Thermo Scientific MaxQ 6000, Thermo Fischer Scientific, Waltham, MA, USA) at 37 °C for 12 h, under stirring at 150 rpm. Extracts were transferred into a 50 mL volumetric flask, and the volume was adjusted with Milli-Q water. Samples were then filtered through Whatman No 40 filter paper. Sodium borohydride solution (4.5 mL at 0.1 mol/L) was added to 5.0 mL of the filtrate mixture, mixed gently for 30 s, then 0.5 mL of glacial acetic acid was added and mixed for 30 s. For HPLC analysis, samples were filtered through a 0.45 µm PTFE filter (VWR, Radnor, PA, USA). Total vitamin B6 was analyzed on an Agilent HPLC system (1260 Infinity, Agilent Technologies, Santa Clara, CA, USA) coupled to a fluorometric detector, operating in an isocratic mode. The mobile phase consisted of methanol with 0.01 mol/L phosphoric acid (26:74, *v:v*) at pH 2.5. Separation was performed on a Luna C18 150 × 4.6 mm, 5 µm column (Phenomenex, Torrance, CA, USA), equipped with a guard column (Phenomenex, Torrance, CA, USA). The flow rate was 1.0 mL/min for 55 min, the oven temperature was 25 °C, and the injection volume was 50 μL. Quantification was performed with external calibration against pyridoxine as total vitamin B6 [27]. The limit of detection and limit of quantification were determined as 0.06 μg/mL and 0.19 μg/mL, respectively.

### 2.4. Moisture Content Determination

Moisture content was determined by calculating the ratio between the mass before and after drying. Briefly, 0.50 g of each sample was weighed and then directly placed in an oven (Lindberg Blue M, Thermo Scientific, Waltam, MA, USA). Samples were heated at 100 °C overnight, and then removed from the oven, placed in a desiccator, cooled to room temperature, and weighed.

### 2.5. Theoretical Consideration and Statistics

#### 2.5.1. Reaction Kinetics

Vitamin degradation was modeled using the first-order kinetics Equation (1) as described for other water-soluble vitamins [21].

(1) $${C_{t}=C}_{0}e^{\left(-kt\right)}$$

where C_t_ is the concentration at time t in seconds, C_0_ = 1 is the initial (normalized) concentration, and k = k(T) is the rate constant, which depends on the temperature T.

The activation energy was determined according to the Arrhenius law:

(2) k=eEaRT

where k is the rate constant, $E_{a}$ is the activation energy, R is the ideal gas constant, and T is the temperature in Kelvin.

#### 2.5.2. Diffusion

The diffusion of vitamin B6 in chickpeas was modeled using Fick’s second law, utilizing the simplified integration for spherical shape.

The general equation for Fick’s second law is:

(3) $$\frac{\partial C}{\partial t}=-D\left(T\right)\frac{\partial C^{2}}{\partial r^{2}}$$

where *C = C(r,t)* is the concentration of the molecule under consideration at time t (in seconds) and at distance r (in meters) from the center of the sphere, and D = D(T) is the diffusion constant, which depends on the temperature T.

The solution of Equation (3) for spheres is given by Crank (1975) [28], assuming an initially uniform distribution of the vitamins in the plant material:

(4) $$C_{t}=C_{eq}+(C_{0}-C_{eq})\frac{6}{\pi^{2}}\sum_{n=1}^{\infty }\frac{1}{n^{2}}\exp(-\frac{D\left(T\right)\pi^{2}n^{2}t}{R^{2}})$$

Here, $C_{eq}$ is the equilibrium concentration, which would be reached if the vitamins were present at the same concentration in all compartments, and *R* is the radius of the sphere in meters.

The shell of a typical chickpea is not a perfect sphere but a prolate spheroid. Thus, the middle radius (i.e., equatorial radius), denoted as a, is smaller than the radicle radius (i.e., polar radius), denoted as c. Since the amount of diffusion depends primarily on the surface area of an object, we performed the following mathematical procedure that allows us to assume that chickpea shells are spheres. For 50 chickpeas, we measured the middle radius and radicle radius and computed the surface area of each chickpea, given as follows:

(5) $$S_{\mathrm{prolate}\;}\left(a,c\right)=2\pi a^{2}+2\pi \frac{ac}{e}{sin}^{-1}\left(e\right)$$

where $e=\sqrt{1-\frac{a^{2}}{c^{2}}}$ is the ellipticity of the prolate spheroid. For a sphere, *a = c* so that the surface area of a sphere with radius *R* is as follows:

(6) $$S\left(Rr\right)=4\pi {Rr}^{2}$$

With this, we derived the radius R of a sphere for each chickpea, with the same surface area as the chickpea spheroid. That is,

(7) $$R\left(a,c\right)=\sqrt{\frac{S_{\mathrm{prolate}\;}(a,c)}{4\pi }}$$

Given the middle radius measurements *a_i_* and the radicle radius measurements *c_i_* for 50 chickpeas, we inferred the mean radius of the sphere with an equal surface area and used this to compute the degradation and diffusion constants, as shown below:

$$R=\frac{1}{50}\sum_{i=1}^{50}r\left(a_{i},c_{i}\right)=4.6143\;mm,$$

As degradation and diffusion happen at the same time, we solved diffusion Equation (8) with first-order degradation as follows:

(8) $$\frac{\partial C}{\partial t}=-D\left(T\right)\frac{\partial C^{2}}{\partial r^{2}}-k\left(T\right)C$$

Similar to Crank (1975) [28], the solution is given by the following Equation (9):

(9) $$C_{t}={\exp\left(-k\left(T\right)t\right)[C}_{eq}+(C_{0}-C_{eq})\frac{6}{\pi^{2}}\sum_{n=1}^{\infty }\frac{1}{n^{2}}\exp(-\frac{D\left(T\right)\pi^{2}n^{2}t}{a^{2}})]$$

The sum in Equation (9) was developed up to n = 100, and the equation contains both the rate constant k(T) and the diffusion constant D(T).

Fitting both constants simultaneously is not feasible due to their high correlation (linked to the acceleration of degradation and diffusion with increased temperature) and the limited number of data points. The solution (Equation (9)) decreases as k(T) increases and as D(T) increases. For this reason, and due to low sample size, one can find many good fits to the time series, with the specific choices for k(T) and D(T) varying widely between these fits. In this study, we are particularly interested in the specific k(T) and D(T) values and not the absolute best fit for predictive purposes. Therefore, we deemed it not prudent to fit two collinear parameters simultaneously. While the fit would be good (i.e., marginally better than just fitting one at a time), the results would be uninterpretable. Therefore, we opted to run two separate experiments, one with thermal degradation only and one that also allows for diffusion. We obtained best-fit values for the rate constants k(T) from the first experiment following the modelling described in Section 2.5.1. These estimated rate constants are then assumed as the ground truth when deriving the best-fit value for D(T) from the second experiment [21].

#### 2.5.3. Statistics

All degradation and diffusion experiments were carried out in triplicate. Model fitting was performed in Python 3.10.9 by minimizing the sum of squares of the differences between predicted and observed concentrations *C_t_*. Given the nonlinear nature of the models and the slightly varying number of observations per time series, the goodness of fit was evaluated using Mean Squared Errors (MSE). We averaged the rate constants, k(T) and D(T), using the geometric mean in triplicate.

The dispersion of vitamin content in the raw material is described using boxplots (24 replicates for the total raw material and 12 for each condition, diffusion or thermal treatment).

A two-way ANOVA was performed using JMP Pro 16 (SAS Institute Inc., Cary, NC, USA), examining whether treatment (thermal or thermal plus diffusion) and/or temperature or the interaction between the two variables significantly impact the residual vitamin B6 concentration. Similar statistical analysis was performed to determine if significant differences existed in moisture content among samples at the 4 temperatures for the diffusion over the 4 h of the kinetics. For all ANOVAs, significant differences were determined using a post hoc Tukey HSD test, with a 0.05 significance level (*p* < 0.05).

## 3. Results and Discussion

### 3.1. Initial Content of Vitamin B6

**Figure 1:**
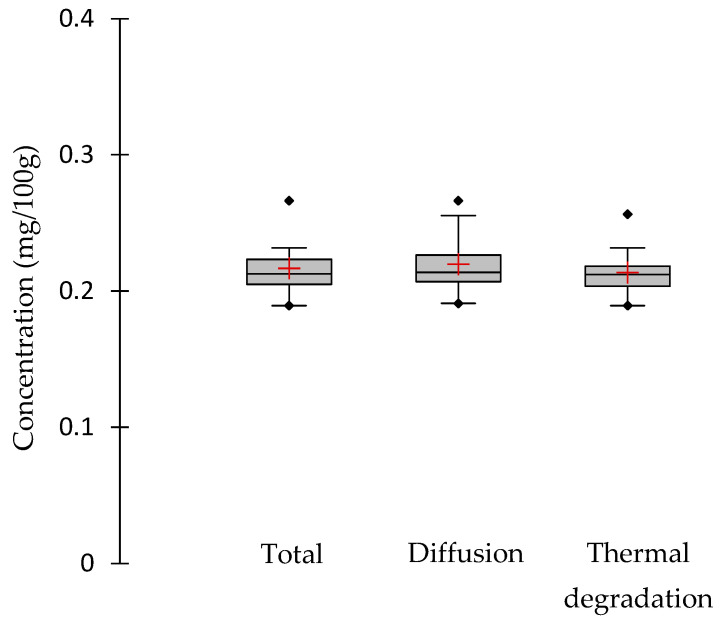
Vitamin B6 variability in raw chickpeas. The boxplots represent the variability in the concentrations in all initial materials (total) in initial samples used for the diffusion kinetics (diffusion) and the thermal degradation kinetics (thermal degradation) at time t0, at the beginning of the experiments. Red crosses represent the average of the concentrations, and rhombus the maximum and minimum concentrations.

Initial vitamin B6 concentrations (after pre-treatment of 30 min in boiling water) varied from 0.189 mg/100 g to 0.266 mg/100 g, with an average initial concentration of 0.217 mg/100 g (Figure 1). The variability of the initial vitamin B6 concentration in samples was very low compared to other water-soluble vitamins, such as folate or vitamin C [21], and is in agreement with previous data [3,7,17]. All kinetics were performed using similar initial batches. At the same time, the moisture content of the samples varied from 30.4 ± 8.3%, for initial samples used in the diffusion kinetics at 85 °C, to 47.4 ± 0.9%, for initial samples used in the thermal degradation experiment at 65 °C. No significant difference existed between the moisture content of the initial samples, according to a Tukey test (Table 1). The preliminary 30 min boiling treatment allowed for hydration of the legumes, disruption of plant tissues, and inactivation of endogenous enzymes, so that the water-soluble vitamin could diffuse freely. This preliminary treatment did not induce significant variability in the initial vitamin concentration or moisture content.

### 3.2. Thermal Degradation Kinetics

**Figure 2:**
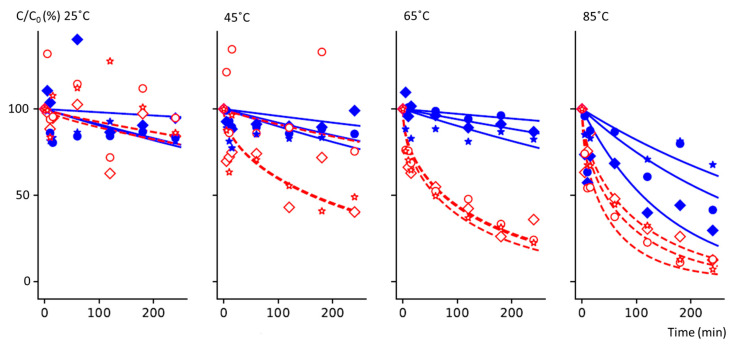
Thermal degradation and thermal degradation plus diffusion of vitamin B6 in chickpeas at 25, 45, 65, and 85 °C. Blue lines represent the model-predicted thermal degradation (samples sealed in plastic bags), and blue full circles, stars, and diamonds represent the observed data from the three replicates. Red dashed lines represent the model-predicted thermal degradation when allowing for diffusion; empty red circles, stars, and diamonds represent the observed data from the three replicates. The best-fit model parameters are presented in Table 2.

Thermal degradation kinetics were carried out in hermetic bags to prevent loss of vitamins by diffusion. Best-fit curves for the first-order modeling of changes in vitamin B6 content at eight different time points are shown in Figure 2 (blue solid lines). Table 2 presents the residual content (after 240 min) and the kinetic constant k at each temperature (geometric mean across the replicates).

Vitamin B6 did not notably degrade at 25, 45, or 65 °C, with degradation ranging from 10 to 14% after 4 h of treatment (Table 2). Moreover, the variability of the concentrations after 4 h between the three individual kinetics remained low, with a coefficient of variation (CV) of 8.2% at 25 °C, 8.7% at 45 °C, and 4.2% at 65 °C (Figure 2). However, at 85 °C, the degradation and the variability between the three kinetics were more pronounced, with a residual content of 46.3% (53.7% degradation) and a CV of 42%, respectively (Figure 2). The moisture content did not change significantly across the four hours of treatment, implying that our experiments were carried out in a complete hermetical system, i.e., that dilution did not affect the diffusion of the vitamin (Table 1).

Reliable degradation modeling using a first-order reaction law requires at least 70% loss [29]. Since loss did not exceed 55%, the kinetic data did not favor using a first-order reaction law for the thermal degradation of vitamin B6, which exhibits a limited degradation level (Table 2). The second data modeling approach would have been to use a pseudo-first-order with a plateau, as previously described [30]. However, the data did not suggest the appearance of a plateau during the thermal degradation kinetics (Figure 2). Therefore, we utilized a first-order modeling approach for the thermal degradation of the vitamins, as reported previously for other water-soluble vitamins [19,21,30], even if this approach has some limitations.

The reaction rate was about five times higher at 85 °C (54.9 × 10^−6^ s^−1^) than at 25, 45, and 65 °C (9.35 to 11.8 × 10^−6^ s^−1^) (Table 2). Temperature did not affect the thermal degradation of the vitamin: the activation energy $E_{a}$ was calculated as 21.7 × 10^3^ J/mol with an R^2^ of 0.59. Even though the correlation coefficient is low, the calculated $E_{a}$ is still in agreement with ones calculated for vitamin C, ranging from 34.2 × 10^3^ J/mol to 60.8 × 10^3^ J/mol in green peas, but lower than one calculated for folates [21,31]. This lower $E_{a}$ could be explained by a more complex chemical structure for folate than pyridoxine. Also, the nature of the food matrix and its macromolecule composition could affect molecule stability and, consequently, the kinetic parameters [32,33].

**Table 2:** Vitamin B6 residual content, first-order reaction constants and effective diffusion constants.

Temperature (°C)	Residual Content (%) *		k (s^−1^)	MSE	D (m^2^/s)	MSE
	Thermal	Diffusion				
25	86.6 ± 7.1	88.5 ± 5.6	9.35 × 10^−6^	1.52 × 10^−2^	4.76 × 10^−14^	2.25 × 10^−2^
45	90.0 ± 7.9	55.0 ± 18.3	11.8 × 10^−6^	7.01 × 10^−3^	3.13 × 10^−12^	2.75 × 10^−2^
65	86.3 ± 3.6	27.2 ± 6.7	9.38 × 10^−6^	2.64 × 10^−3^	1.60 × 10^−10^	4.58 × 10^−3^
85	46.3 ± 19.5	10.9 ± 3.3	54.9 × 10^−6^	2.26 × 10^−2^	2.07 × 10^−10^	5.34 × 10^−3^
F-value thermal vs. diffusion		33.8				
*p*-value		<0.0001				
F-value temperature		61.5				
*p*-value		<0.0001				
F-value interaction between treatment and temperature		7.7				
*p*-value		NS				

* Percentage of the relative to initial content—NS: not significant. F-value and *p*-value from a two-way ANOVA.

Vitamin B6 seems to be more stable when exposed to heat than expected. This is in agreement with prior research on folates, water-soluble vitamins found in fruits and vegetables, which describe a high stability of the folates during hydrothermal treatments, as well as on cooked meat, where non-significant degradation of vitamin B6 was determined especially for long treatments [19]. The potential protection of the food matrix and interactions of the vitamin with other macromolecules, such as proteins, could explain the low degradation of vitamin B6 in the studied conditions.

### 3.3. Diffusion Kinetics

In contrast to thermal degradation, losses due to diffusion were pronounced, especially at high temperature. Vitamin B6 residual content varied from 10.9% at 85 °C to 88.5% at 25 °C (Table 2), with a high impact of the temperature (r = 0.95, *p* < 0.001). Time curves for the diffusion model are presented in Figure 2 (red dashed lines). Moreover, the diffusion variability between the three independent kinetics was low but increased depending on the temperature, with a marked effect at 85 °C.

**Figure 3:**
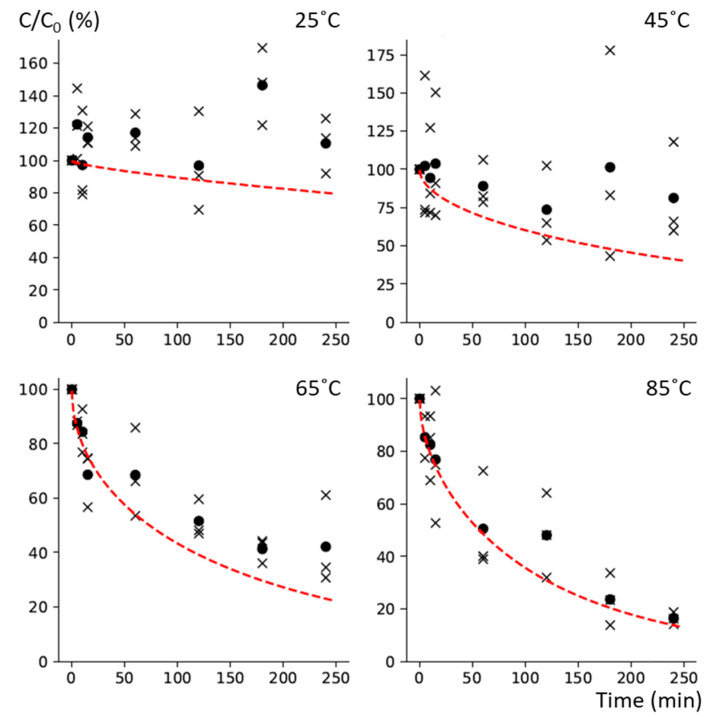
Evolution of the normalized concentration of the vitamin B6 for diffusion kinetics, expressed in dry weight. Black x represents the normalized concentration level, C/C_0_, for each individual kinetics, and the solid black circles represent the average of the triplicate; the red dashed line represents the mean of the model prediction (diffusion + thermal degradation) from Figure 2.

Moisture content in chickpeas did not increase significantly during diffusion kinetics experiments at 25, 45, and 65 °C, but was significantly higher at t_240_ when compared to t_0_ (*p* = 0.0098) for the kinetics at 85 °C (Table 1). Even if the intake of water during the diffusion kinetics, especially after 4 h, was significant, the effect of the dilution did not seem to influence the diffusion, as shown by plotting the evolution of the vitamin B6 concentration expressed in dry matter (Figure 3). Therefore, regarding the focus of our study on the impact of processing on the nutritional quality of legumes, we have decided to express our results in terms of fresh weight, which is more representative for our purpose.

Diffusion kinetics were modelled according to an adapted Fick’s second law, as detailed in Section 2.5.2. The modeling results are presented in Figure 2, and the estimated diffusion constants and mean square errors are shown in Table 2. The diffusivity constant varied from 4.76 × 10^−14^ m^2^/s to 2.07 × 10^−10^ m^2^/s. Temperature tended to impact the diffusivity constant (r = 0.94, *p* = 0.059), though not significantly. There was variance in a factor of 100 for the kinetics at 25 °C, 45 °C, and 65 °C. In contrast, above 65 °C, the temperature seemed to have less of an effect, with only a factor of two between the diffusivity constant calculated at 65 °C and 85 °C. The diffusivity constant calculated at 25 °C was in the same range (8.9 × 10^−14^ m^2^/s and 4.4 × 10^−13^ m^2^/s) as those calculated at a similar temperature (23 °C) in an agarose–gelatin model system [26]. Moreover, the diffusivity constants calculated for vitamin B6 at 65 °C and 85 °C were in agreement with those calculated for folates and vitamin C in Brussels sprouts and peas, respectively, but ten times lower than those calculated for folates in peas at the same temperatures, which were 8.1 × 10^−11^ m^2^/s at 65 °C and 8.8 × 10^−11^ m^2^/s at 85 °C. The diffusivity of vitamin B6 appeared to behave more similarly to vitamin C than folates [21,30,34,35]. This observation could be related to the chemical structure of these molecules or molecular weight, which is similar for vitamin B6 and vitamin C, and 2.5 times lower for folates. Also, quantifying the different vitamers might lead to some misevaluation of the diffusivity, as vitamin B6 and folates were quantified after conversion of all the different vitamers into one.

### 3.4. Thermal Degradation Versus Diffusion

Thermal degradation and the diffusion modeling of vitamin B6 during hydrothermal treatment of chickpeas revealed that degradation was mainly due to leaching (diffusion), especially during short treatment times (Figure 2 and Table 2).

The order of magnitude of k (10^−6^ s^−1^) and the order of magnitude of D (ranging from 10^−10^ to 10^−14^ m^2^/s) cannot be compared as such to explain the loss of the vitamin, as they do not represent the same physicochemical mechanisms, and they must be expressed in different units. The diffusivity constant was affected by the temperature (r = 0.94, *p* = 0.059), while the thermal degradation constant was affected to a lesser extent (r = 0.77, *p* = 0.226). Moreover, the residual content of the vitamin was higher when only considering thermal degradation. The type of treatment had less of an effect than temperature, but was still highly significant (Table 2). Diffusion and thermal degradation appeared to be a second factor for the levels of the residual vitamin after 4 h of treatment, confirming the important role of temperature on the residual content. However, the low *p*-value related to the kinetic impact (thermal vs. diffusion) reflects the importance of the diffusion in the loss of vitamin B6 during hydrothermal treatment. This observation is supported by others which showed that blanching or cooking in water leads to a greater loss of water-soluble vitamins (folates and vitamin C) from small vegetables than culinary steaming or industrial blanching for durations of about 10 min or less [36,37]. For vitamin B6, similar results were described for cooked meat [19]; however, fewer results are documented for fruits, vegetables, or legumes.

Modeling the chemical reactivity in foods is challenging because of complex molecular composition and the subcellular compartmentation [38]. Diffusion is rarely considered explicitly when modeling vitamin losses from plant matrices. However, two points seem relevant when comparing the results obtained in hermetically sealed bags versus in an open excess of water. The first one is that higher losses were observed with leaching, and the evolution of the concentrations within the material can be adequately modeled using a first-order kinetic model. The second one is that the two models give somewhat similar line shapes (Figure 2), the difference being that modeling of leaching better explains the fast-observed vitamin losses for short treatment durations (Figure 2).

## 4. Conclusions

This study enabled the development of a quantitative model to better understand the loss of water-soluble vitamins during hydrothermal treatments of chickpeas, which is highly relevant for further improvement of assessing the nutritional value of processed fruits, vegetables and legumes. Vitamin B6 is more stable at high temperatures than previously thought, with a decrease in the content only observed at 85 °C. Diffusion is the main factor driving vitamin B6 loss during cooking, especially at shorter cooking times. During hydrothermal treatment, temperature greatly impacts both the residual content of vitamin B6 and the diffusivity constant. The present study, performed with chickpeas as a food model, can potentially be adapted to other food systems sharing similarities in terms of shape and structure, especially ones rich in protein to further advance our understanding of diffusion and chemical degradation of vitamins in foods.

## Figures and Tables

**Table 1:** Moisture content of cooked chickpeas subjected to diffusion and thermal degradation kinetics (in %).

Time (min)	Thermal Degradation				Diffusion				
	25 °C	45 °C	65 °C	85 °C	25 °C	45 °C	65 °C	85 °C	
0	40.6 ± 3.9	43.7 ± 6.8	47.4 ± 0.9	39.8 ± 3.9	38.7 ± 8.0	43.9 ± 4.7	47.2 ± 3.9	30.4 ± 8.3	a
5				36.7 ± 6.2	45.1 ± 5.4	46.0 ± 9.3	54.2 ± 3.1	nd	
10				34.2 ± 2.5	42.3 ± 9.1	55.1 ± 2.9	55.6 ± 3.8	44.7 ± 5.8	abcdef
15				37.1 ± 3.3	46.7 ± 6.8	43.6 ± 1.7	49.0 ± 7.1	34.8 ± 16.0	ab
60				38.6 ± 7.5	42.5 ± 7.1	51.4 ± 1.1	58.7 ± 7.1	36.7 ± 16.0	abc
120				53.8 ± 4.2	45.6 ± 5.7	52.7 ± 5.3	56.6 ± 4.3	56.5 ± 12.3	cdef
180				44.4 ± 2.4	56.4 ± 5.8	52.4 ± 2.0	61.3 ± 2.0	50.6 ± 10.8	abcdef
240	41.5 ± 3.4	45.7 ± 2.3	50.8 ± 1.8	50.9 ± 6.2	50.6 ± 6.5	61.7 ± 1.5	64.9 ± 3.5	54.7 ± 7.9	bcdef

Data are presented as mean ± standard deviation—nd: not determined. ^abcdef^ statistically significant differences (*p* < 0.05 after post Tukey HSD correction) exist between different time points whenever letters are not shared. Time = 5 min excluded from statistical analysis due to a missing data point.

## Data Availability

The original contributions presented in the study are included in the article, further inquiries can be directed to the corresponding author.
